# *Daphnia magna’s* sense of competition: intra-specific interactions (ISI) alter life history strategies and increase metals toxicity

**DOI:** 10.1007/s10646-016-1667-1

**Published:** 2016-05-05

**Authors:** Kurt A. Gust, Alan J. Kennedy, Nicolas L. Melby, Mitchell S. Wilbanks, Jennifer Laird, Barbara Meeks, Erik B. Muller, Roger M. Nisbet, Edward J. Perkins

**Affiliations:** Environmental Laboratory, US Army, Engineer Research and Development Center, Vicksburg, MS USA; SpecPro Technical Services, San Antonio, TX USA; Marine Science Institute, University of California, Santa Barbara, Santa Barbara, CA USA; Department of Ecology, Evolution & Marine Biology, University of California, Santa Barbara, Santa Barbara, CA USA

**Keywords:** *Daphnia*, Intra-specific interactions, Ecotoxicology, Metals toxicity, Standard toxicity assays

## Abstract

This work investigates whether the scale-up to multi-animal exposures that is commonly applied in genomics studies provides equivalent toxicity outcomes to single-animal experiments of standard *Daphnia magna* toxicity assays. Specifically, we tested the null hypothesis that intraspecific interactions (ISI) among *D. magna* have neither effect on the life history strategies of this species, nor impact toxicological outcomes in exposure experiments with Cu and Pb. The results show that ISI significantly increased mortality of *D. magna* in both Cu and Pb exposure experiments, decreasing 14 day LC50 s and 95 % confidence intervals from 14.5 (10.9–148.3) to 8.4 (8.2–8.7) µg Cu/L and from 232 (156–4810) to 68 (63–73) µg Pb/L. Additionally, ISI potentiated Pb impacts on reproduction eliciting a nearly 10-fold decrease in the no-observed effect concentration (from 236 to 25 µg/L). As an indication of environmental relevance, the effects of ISI on both mortality and reproduction in Pb exposures were sustained at both high and low food rations. Furthermore, even with a single pair of *Daphnia*, ISI significantly increased (p < 0.05) neonate production in control conditions, demonstrating that ISI can affect life history strategy. Given these results we reject the null hypothesis and conclude that results from scale-up assays cannot be directly applied to observations from single-animal assessments in *D. magna*. We postulate that *D. magna* senses chemical signatures of conspecifics which elicits changes in life history strategies that ultimately increase susceptibility to metal toxicity.

**Electronic supplementary material:**

The online version of this article (doi:10.1007/s10646-016-1667-1) contains supplementary material, which is available to authorized users.

## Introduction

*Daphnia magna* is an important model species in ecotoxicology for which standard assays have been developed for use in regulatory toxicity assessment (ASTM 2012; USEPA 2002). Over the past 10 years, *Daphnia* spp. have become increasingly utilized as genomic model organisms (Colbourne et al. 2011) and used in toxicogenomic investigations to determine molecular and mechanistic effects of contaminant exposures (Ananthasubramaniam et al. 2015). In order to meet minimum mRNA requirements for toxicogenomics methods, a common practice has been to scale up exposures to include 10–100 s of *Daphnia* per exposure replicate. This type of scale-up procedure has been applied in a number of toxicogenomics studies with *D. magna* (i.e. Stanley et al. 2013; Campos et al. 2013; Garcia-Reyero et al. 2009, 2012; Poynton et al. 2007; Shaw et al. 2007) where the expression results were directly applied to understand the results observed in standard-scale, single animal exposures. In order to draw these inferences among exposure methods, the authors have made the assumption that exposure scaling has no effect on the outcome of the test. Thus far, we have found no published studies that have explicitly tested this critical assumption for *D. magna* in ecotoxicological exposures, in context with genomics investigations.

When scaling up a toxicity assay such as the standard *D. magna* reproduction test (ASTM 2012) from a single animal to multi-animal exposure experiment, it becomes logistically challenging to quantify reproductive output. Note that the cited ASTM method allows the experimentalist discretion to test either signal or multiple organisms within a single replicate chamber. Thus, many researchers run scale-up toxicity assays in parallel, where the latter is used to quantify reproduction and the former to examine toxicogenomic effects. However, animals exposed in the scale-up tests could potentially experience intra-specific competition, whereas individuals in the standard assays would not. The adverse effects of contaminant exposure on survival, reproduction and/or population structure can be exacerbated by intraspecific competition among *Daphnia* (Knillmann et al. 2012; Foit et al. 2012; Liess and Foit 2010; Viaene et al. 2015). In this context, researchers are often careful to scale up both exposure volume and food per individual to help minimize the potential for intraspecific competition among *Daphnia* in the multi-animal exposures, thus minimizing confounding effects of intraspecific competition on toxicity in scale-up assays.

Nonetheless, intraspecific competition may not be the only potential confounding factor that can be introduced when scaling up from single animal to multi-animal exposures, no matter how carefully designed to standardize food and exposure volume between assays. For example, intraspecific interactions (ISI) may elicit effects that are independent of the two primary drivers of intra-specific competition (population density and resource availability), and cause significant changes in life history trajectories, resource allocation and reproduction (Hobæk and Larsson 1990; Burns 1995; Lürling et al. 2003; Burns 2000). In these studies, *Daphnia*-conditioned water was produced by culturing *Daphnia* under crowding stress and naïve individuals were then exposed to the conditioned water resulting in changes in life history strategies including increases in body size, body length and brood size. These observations suggest that, in response to crowding, *Daphnia* releases chemical cues (i.e. metabolites or pheromones) that alter the behavior and life history strategies of conspecifics. Accordingly, we posit that ISI can occur in *Daphnia* that are not specifically the result of density-dependent competition, but are rather due to the ability of *Daphnia* “to sense” conspecifics and change behavior and life history strategies. Given this phenomenon, we suspect that ISI-initiated effects on *Daphnia* life history strategies could confound toxicity results in scale-up assays with *D. magna,* even when methods are applied to minimize intra-specific competition.

Given the current practice of using scale-up assays to investigate the molecular basis of effects seen in single-animal standard assays in *D. magna* without considering the potential impacts of intraspecific competition and ISI on behavior and life history strategies, it is critical to test the assumption that scaling up to multi-individual exposure experiments has no effect on toxicity outcomes. Therefore, our objective was to explicitly test this assumption using standard survival and reproduction endpoints in *D. magna*. We exposed *D. magna* to Cu and Pb, which elicit toxicity responses in *D. magna* via unique mechanisms of action (Roy 2009; Poynton et al. 2007) and assessed if ISI modulated survival and neonate production. Specifically, we tested the null hypothesis that ISI among *D. magna* affect neither the life history strategies nor the toxicological outcomes of Cu and Pb exposure experiments.

## Materials and methods

### Experimental animals and general exposure methods

*Daphnia magna* neonates (<24 h old) were obtained from in-house cultures within the Environmental Laboratory (US Army, Engineer Research and Development Center), originally purchased from Aquatic Biosystems (Fort Collins, CO, USA; original source was EPA Ohio, AROF2, Lot No. 070092DM) in May of 2009 from which a single individual was used to initiate in-house cultures to ensure testing in a single clone. All culturing and test methods used moderately hard reconstituted water (MHRW), formulated according to USEPA (2002). Assays were static renewal, with freshly prepared media supplied three times per week. All experiments were conducted at 25 ± 1 °C, with a photoperiod of 16 h light to 8 h dark (Darwin Environmental Chambers, St. Louis, Missouri, USA). Exposure methods were adapted from the Organisation for Economic Cooperation and Development method 202 (OECD 1984) where animals were exposed in 14 day assays and fed only algae. In a parallel effort, the effects of various feeding rations (provided in the supplemental text to provide context for our selected feeding method) on life history strategy were assessed. Water quality parameters (temperature, pH, dissolved oxygen, specific conductivity) were recorded for both in- and out- water through the course of all assays where all values remained within the standard range of acceptability.

### Effect of intra-specific interactions (ISI) on metal toxicity

To assess if ISI introduced during exposure scale-up from single- to multi-animal exposures effected toxicity outcomes in *D. magna*, we simultaneously conducted standard single animal assays (OECD 1984) and proportionally-scaled assays with 20 animals per exposure replicate. *D. magna* were exposed to the model toxicants Cu and Pb where the effects of Cu on survival were assessed while the effects on both survival and reproduction were assessed for Pb. The Cu (CuSO_4_, 7758-98-7, 98.2 %, Fisher Scientific, Fair Lawn, New Jersey, USA) and Pb (PbCl_2_, 7758-95-4, 99.999 %, Aldrich Chemical Company, Inc., Milwaukee, Wisconsin, USA) treatments consisted of exposures to five concentrations of either Cu (nominally: 0, 2, 4, 6, 9, and 12 µg/L) or Pb (nominally: 0, 11, 21, 42, 84, 168 µg Pb/L) using MHRW as the dilution and control water. The standard test used a single *D. magna* neonate (<24 h old) exposed in 50 mL glass beakers (5.6 cm tall with 4.2 cm diameter) containing 40 mL MHRW, replicated eight times in the Pb exposure or fifteen times in the Cu exposure. The standard assay was tested against the scale-up treatment, where 20 *D. magna* neonates (<24 h old) were exposed in 1000 mL glass beakers (15 cm tall with 11 cm diameter) containing 800 mL MHRW, with six replicates for both the Pb and Cu exposures. Both methods provided an equal water volume per daphnid (40 mL/individual) and equal daily rations (per unit volume) of green algae (*R. subcapitata*) per initial neonate number. The resulting food- and volume-normalized experimental design provided the ability to explicitly test the effects of ISI on critical toxicity endpoints. We were additionally interested in determining if food levels influenced ISI and resultant effects on toxicity outcomes, therefore in the Pb exposure we included two feeding ration treatments of lower (1.8 × 10^5^ cells/mL) and higher (3.6 × 10^5^ cells/mL) relative food levels (see supplemental text and Supplemental Fig. S1 for rationale on food levels). Lethality was determined at day 14 for both the Cu and Pb exposures. Neonate production in the Pb exposures was determined at two time points to assess reproduction at important life stages for *Daphnia*: (1) post-first brood (day 9) and (2) post-third brood (day 14). Destructive sampling was employed to quantify reproduction in the scale-up treatments (20 *Daphnia*/1000 mL beaker), therefore the results for the 9d and 14d sampling periods represent independent measures as opposed to repeated measures. Reproduction results were standardized as mean cumulative neonates per surviving adult.

### Analytical chemistry

Water samples (2 mL) were taken from each assay concentration throughout the tests, preserved with nitric acid (3 % v/v) and Pb and Cu concentrations determined by inductively-coupled plasma mass spectrometry (Elan DRC-II, PerkinElmer, Waltham, MA) according to standard EPA 6000/7000 series methods (EPA method 6020 and 6010B). The reporting limit ranged from 0.2 to 0.5 ppb depending on the test and sample dilution factor (for both Cu and Pb). Concentrations remained relatively stable and the arithmetic mean of each exposure concentration was used for endpoint calculations.

### Assessing the threshold for ISI effects

An additional assay in the absence of chemical exposure was conducted to test the effects of increasing degrees of ISI on reproduction. The assay utilized the same ratio of MHRW volume to number of organisms (40 mL MHRW per organism) as well as a constant food ration of green algae (*R. subcapitata*) standardized to the number of *D. magna* in each chamber across treatments. Exposure chambers consisted of 50, 100, 250, 600, and 1000 mL glass beakers containing 40, 80, 200, 400, and 800 mL of MHRW, respectively. In order to test the effects of increasing ISI, 1, 2, 5, 10 or 20 individuals, respectively were added to exposure chambers with each treatment including five replicates. By design, initial animal density per unit volume and food ration per individual was equivalent across treatments. The rationale for increasing ISI with increasing number of individuals per exposure chamber was defined as an increased potential for intraspecific interaction with unique conspecifics as the number of individuals per chamber increased. Reproduction and survival were assessed daily throughout the assay and dead individuals were removed from the test beakers. Testing continued until three broods of neonates were obtained for each of the beaker sizes, which in this case took 15 days. Endpoints evaluated at termination of the assays included reproduction, survival, and carapace length, which was calculated using Leica DFC425 (Leica Microsystems Ltd., Heerbrugg, Switzerland) paired with Image-Pro Plus software version 7 (Media Cybernetics, Inc., Bethesda, MD).

### Statistical analysis

All statistical analyses including determinations of data normality (Kolmogorov–Smirnov test) and homogeneity of variance (Levene’s test) were performed using Sigmastat v3.5 software (SSPS, Chicago, IL, USA). Statistical analysis of the full design for each experiment was conducted as described in the following: two-way analysis of variance (ANOVA) for survival in the Cu exposure (Cu concentration X ISI) and three-way ANOVAs for survival and reproduction in the Pb exposures (Pb concentration X ISI X feeding ration). When the assumptions of ANOVA were not met before or after data transformations (arcsin square root or ranking), one-way ANOVAs or non-parametric Kruskal–Wallis one-way ANOVA on ranks were performed with Holm-Sidak multiple-comparisons to identify differences in responses (survival or reproduction) relative to respective controls across the Pb exposure range (Supplemental Fig. S2). An identical approach was then used to determine differences in responses related to ISI and feeding ration within each metal exposure condition (i.e. within controls and for each individual metal-exposure concentration, Supplemental Fig. S2). The *p* value for all tests was set at p = 0.05. Finally, lethal median concentrations (LC50), no observable effect concentrations (NOEC), lowest observable effect concentrations (LOEC) and median inhibition concentrations (IC50) were calculated using ToxCalc (ToxCalc 5.0, Tidepool Scientific Software, McKinleyville, CA, USA).

## Results

### Analytical chemistry

Measured Pb levels closely matched target concentrations for in-water samples (13 ± 3, 25 ± 5, 56 ± 7, 113 ± 11, 236 ± 20 µg/L) and were relatively stable (0–30 % loss) between water exchanges (13 ± 5, 23 ± 9, 48 ± 15, 107 ± 28, 166 ± 61 µg/L). Measured Cu levels also matched target concentrations (0.9 ± 0.4, 2.6 ± 0.5, 5.4 ± 2.9, 7.3 ± 1.4, 9.2 ± 1.7, 11.5 ± 0.4 µg/L). The Pb and Cu measured concentrations reported above represent means and one standard deviation from each mean. Lead and Cu were not detected in the control or dilution water.

### Analysis of ISI in chemical exposures

The assumptions of normality and/or homogeneous variance were not met for the multi-way ANOVAs conducted in the Cu and Pb exposure tests both before and after various data transformations. Given that the assumptions of the multi-way ANOVAs were not met, we proceeded with one-way ANOVAs (parametric if applicable, otherwise non-parametric) to test the effects of each individual experimental treatment as described in the “Materials and Methods” section and in Supplemental Fig. S2. The ANOVA results reported throughout the remainder of this paper represent the results of the one-way ANOVA tests.

### Effect of ISI on survival in Cu exposures

**Fig. 1:**
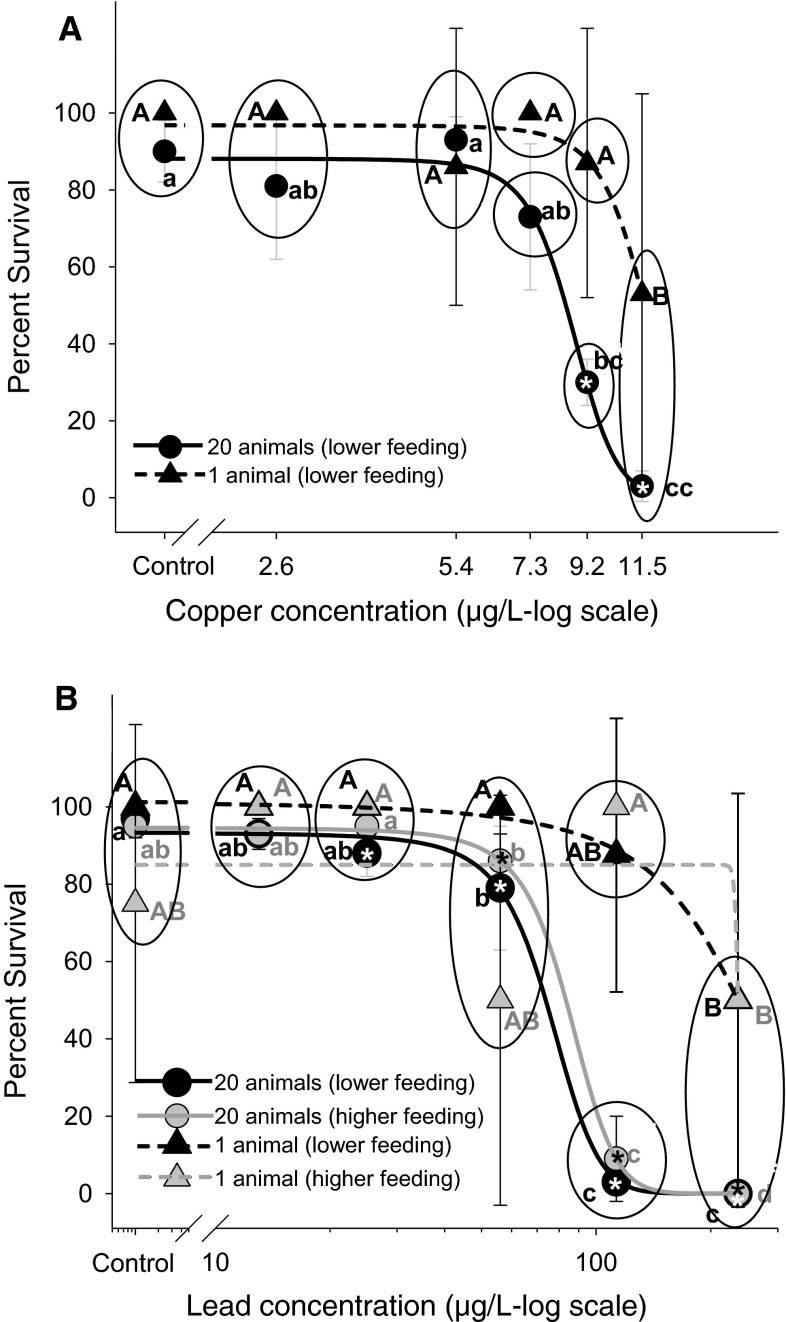
The effect of intra-specific interactions (ISI) on *Daphnia magna* survival in 14 day exposures to Cu (**a**) and Pb (**b**). Effects in the 20 animal treatment (*circular symbols*) were compared to the 1 animal treatment (*triangular symbols*). The influence of lower (*black symbols*) and higher (*grey symbols*) food level in addition to ISI was investigated for Pb. *Symbols* not sharing the *same letter* represent statistically significant differences where *capital letters* refer to the “1 animal” treatment while *lowercase letters* refer to the 20 animal treatment. *Asterisks* denote statistically significant differences relative to the control within each exposure treatment. *Circled groups* of points were not significantly different within each Pb or Cd exposure concentration where the *points* not contained within the *same circle* represent significant differences

The results of the pair-wise tests following one-way ANOVAs indicated significant effects of Cu on *D. magna* survival in each ISI condition, as well as significant differences in ISI effects on Cu toxicity at the 7.3 and 9.2 µg/L exposure concentrations (Fig. 1a). The 14 day LC50 s for the single and group exposure tests were 14.5 µg/L (10.9–148.3, 95 % C.I.) and 8.4 µg/L (8.2–8.7, 95 % C.I.), respectively. The non-overlapping confidence intervals between these LC50 s indicated that the ISI (20 animals exposed together in a single beaker) treatment was significantly more toxic than the non-ISI treatment (1 animal per beaker). It should be noted that the high-end confidence limit for the single *D. magna* test is likely overestimated given the lack of a complete dose–response curve for mortality. This overestimation does not affect the interpretation of the present study, but should be noted for those collecting data for establishing Cu toxicity reference values.

### Effect of ISI and feeding ration on survival in Pb exposures

The results of the pair-wise tests following one-way ANOVAs indicated significant effects of Pb on survival in each ISI× feeding ration condition (Fig. 1b). ISI significantly increased Pb toxicity at the 113 µg/L exposure concentration regardless of low or medium feeding rations (Fig. 1b). The LC50 for Pb decreased by 3 fold in the ISI treatment (68 (63–73) µg/L) relative to the single-animal exposures (232 (156–4810) µg/L) at the lower feeding ration with similar results observed in the higher feeding ration with ISI treatment (LC50 = 79 (74–84) µg/L) and the single-animal no ISI treatment (LC50 = 236 (no 95 % C.I. could be calculated) µg/L. The non-overlapping confidence intervals among treatments indicated that the ISI treatment (20 animals in a beaker) resulted in significantly greater Pb toxicity relative to *Daphnia* that did not experience ISI (single animal), and that these significant effects persisted at two different levels of food availability (Fig. 1b). Similar to the Cu results, the high-end confidence limit for the single *D. magna* test is likely overestimated given the lack of a complete dose–response curve for mortality. Again, this overestimation does not affect the interpretation of the present study, but should be noted for those collecting data for establishing Pb toxicity reference values.

### Effect of ISI and feeding ration on neonate production in Pb exposures

**Fig. 2:**
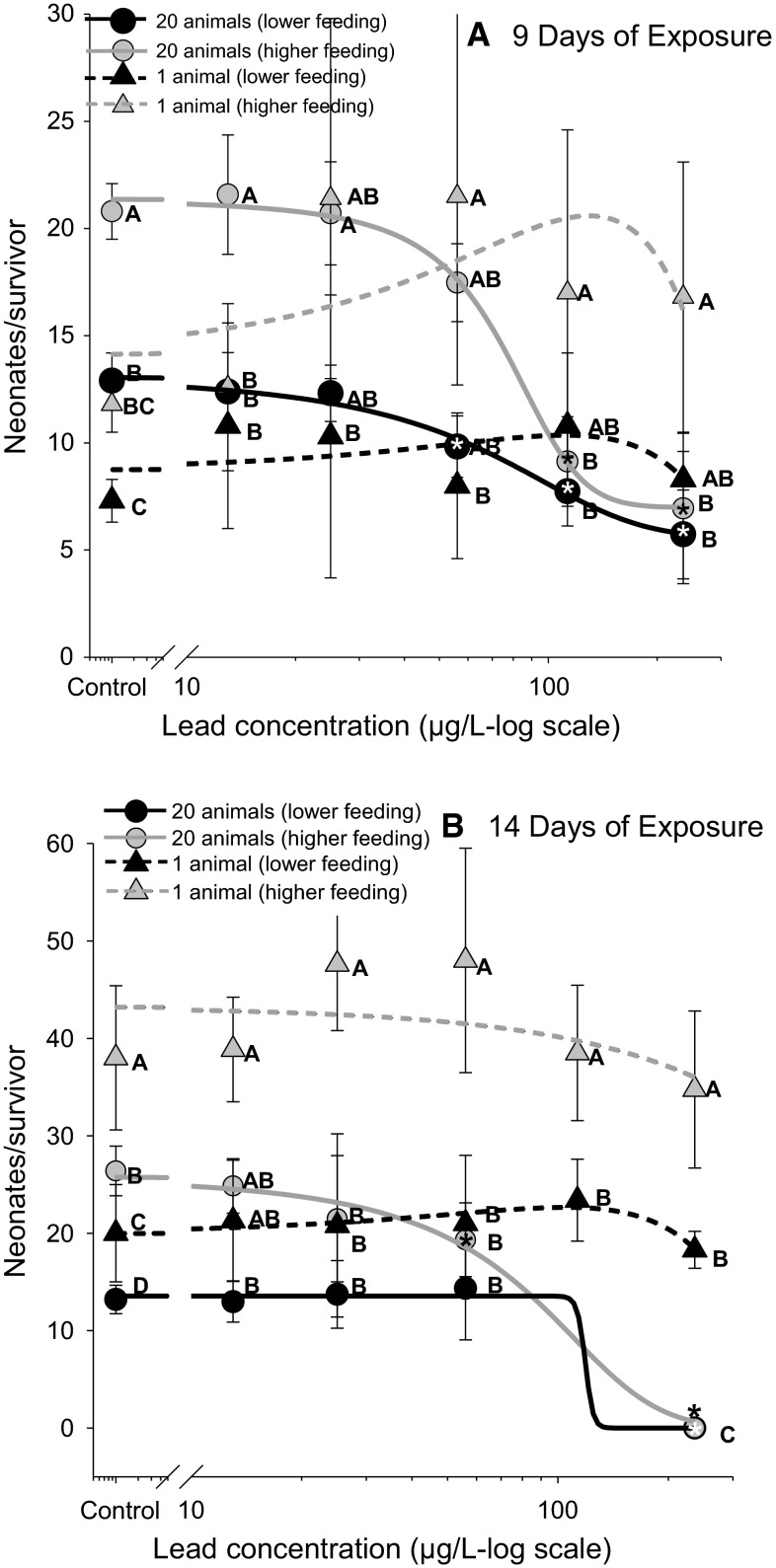
The effect of lead exposure, intra-specific interactions (ISI), and food level on *Daphnia magna* reproduction (neonates per survivor) after first brood (**a** assessed at day 9) and after multiple broods (**b** assessed at day 14). ISI was assessed by keeping food per individual as well as exposure-medium volume equivalent across single animal exposures (“1 animal” per exposure chamber) and multi-animal exposures (“20 animals” per exposure chamber). *Asterisks* denote statistically significant differences relative to the control within each exposure treatment. For example, note the significant difference between the control and the 113 µg/L Pb exposure within the “20 animal (higher feeding)” treatment in **a**. Statistically significant differences between exposure treatments within each lead concentration are represented by differences in *letter* designations. For example, within the 13 µg/L Pb exposure in **a**, reproduction was significantly higher in the “20 animals (higher feeding)” exposure treatment compared to all other exposure treatments

In the 9 day reproductive trial, the results of the pair-wise tests following one-way ANOVAs indicated significant effects of Pb in reducing neonate production in the ISI treatments regardless of feeding ration (Fig. 2a). In contrast, neonate production in the non-ISI treatment was not significantly affected by Pb exposures for either feeding ration (Fig. 2a). The majority of pair-wise comparisons indicated significantly greater neonate production in the higher feeding ration relative to the lower ration. Interestingly, in the 9-d control exposures (0 µg/L, Pb) the ISI treatments resulted in significantly greater neonate production in both the lower and higher feeding rations (Fig. 2a).

As was observed in the 9-d reproductive trial, the results of the pair-wise tests following one-way ANOVAs in the 14-d reproductive trial indicated significant negative effects of Pb exposure on neonate production in the ISI treatment and no significant effects of Pb on reproduction in the treatment without ISI, regardless of feeding ration (Fig. 2b). Additionally, the majority of pair-wise comparisons indicated significantly greater neonate production in the higher feeding ration relative to the lower ration, as was observed in the 9-d reproductive trial. Relative to the observed ISI-induced increase in neonate production in the 9-d controls (Fig. 2a), an inverted response in 14-d reproduction between the ISI and non-ISI treatments was observed in the controls (0 µg/L, Pb); that is, greater neonate production was observed in the single animal exposures in each feeding ration (Fig. 2b).

### Assessing the threshold for ISI effects on neonate production

**Fig. 3:**
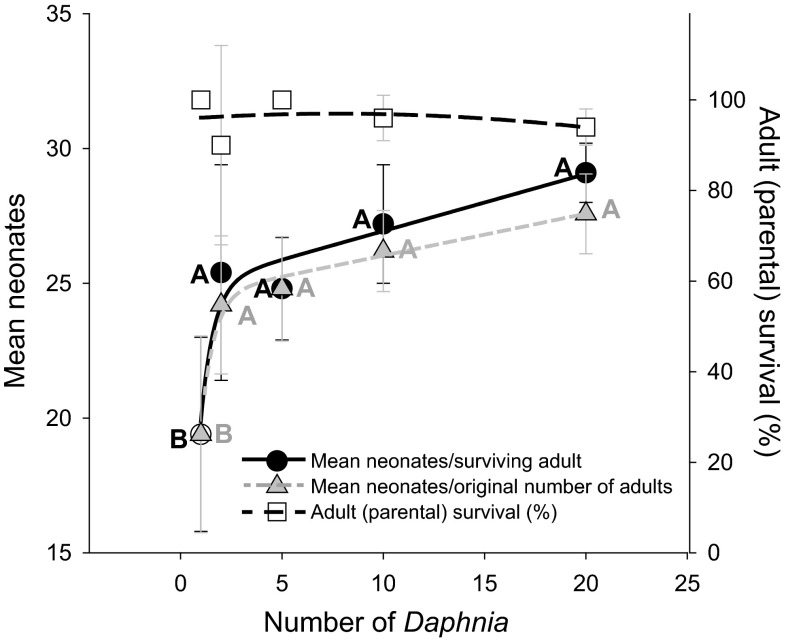
Effect of intra-specific interaction (ISI) on reproduction (neonates per surviving adult) for *Daphnia magna* reared for 15 days in control conditions. The left y-axis presents mean reproduction while the right y-axis presents percent survival of the parental generation. *Bars* represent one standard deviation from the mean. ISI was assessed by keeping food per individual as well as exposure-medium volume equivalent across single animal exposures (1 animal per exposure chamber) and multi-animal exposures (2, 5 10 or 20 animals per exposure chamber). Statistically significant differences in reproduction are represented by differences in *letter* designations (*black letters* denote neonates per surviving adult while *grey letters* denote the total number of neonates without standardization to parental survival). There were no statistically significant differences in parental survival between treatments (p > 0.05)

ISI among *Daphnia* significantly increased reproductive output (p < 0.05, Fig. 3) in the absence of chemical exposure. The threshold for a significant response to ISI occurred at the lowest level of ISI (a pair of *Daphnia* interacting in on beaker) and all higher levels investigated, where significant increases in neonate production were induced. A trend of increasing reproductive output was observed with increasing number of individuals in the exposure chamber; however, the individual data points were not significantly different (Fig. 3).

## Discussion

The experimental results give rise to three primary conclusions with regards to intraspecific interactions (ISI) in *D. magna*. First, the ISI treatment resulted in significantly increased mortality in exposure to both Cu and Pb (Fig. 1) indicating that ISI can increase the lethal sensitivity of *D. magna* to both metals. Second, ISI potentiated the effects of Pb exposure in *D. magna* causing significant decreases in reproductive output compared to no impacts of Pb on reproduction in the absence of ISI (Fig. 2). Third, ISI was observed to alter the critical life history parameter of reproductive output even within control groups, albeit in the opposite direction as observed in the contaminant exposure assays, causing slightly enhanced reproduction (Fig. 3). Further, this response was manifested at the lowest level of ISI (2 *Daphnia* cohabitating a space compared to a single *Daphnia* in isolation). Given these results, we reject the null hypothesis that ISI among *D. magna* has no effect on the life history strategies or the toxicological outcomes of Cu and Pb exposure. Further, the results refute the assumption that experimental scale has no effect on the outcomes of experimental assays conducted with *D. magna*. Below, we discuss the context and potential causes of ISI-mediated increases in sensitivity to Cu and Pb exposure in *D. magna*, the implications of these observations regarding the interpretation scale-up assays applied to observations in standardized toxicity tests, and finally, the implications of ISI in toxicity assessment assays and in the environment.

### Context for ISI-mediated increases in Cu and Pb lethality

Previous observations from both ecology and ecotoxicology literature on intraspecific competition provide valuable context for examining the interactive effects of ISI and contaminant exposure. Regarding effects of contaminant exposure on lethality, both Knillmann et al. (2012) and Foit et al. (2012) observed that increasing intraspecific competition in *Daphnia* increased lethality when exposed to the pesticide esfenvalerate. In these experiments, the density of *Daphnia* was varied prior to contaminant exposure where higher initial densities contributed to increasing intraspecific competition. In contrast, our assays were initiated with equivalent population per unit water volume densities and food per individual across the single and twenty-animal exposures. Thus, the ISI treatment represented a potentially density-independent driver that increased *D. magna’s* lethal sensitivity to the metals Cu and Pb (Fig. 1). Given that esfenvalerate, Cu and Pb elicit toxicity in *Daphnia* by fundamentally different processes (Knillmann et al. 2012; Foit et al. 2012; De Schamphelaere et al. 2007; Poynton et al. 2007; Ananthasubramaniam et al. 2015; Roy 2009), the collective observations of increased lethal sensitivity indicate that ISI within *Daphnia* can exacerbate chemical-induced lethality across multiple modes of toxic action.

### Context for ISI-mediated decreases in reproduction in Pb exposures

Few studies have explicitly investigated the combined effects of intraspecific competition and contaminant exposure on reproduction in *Daphnia.* Existing studies have primarily examined population structure in the contaminant exposures. For example, intraspecific competition in *D. magna* was observed to delay the recovery of population structure long after contaminant exposure, as was demonstrated in exposures to fenvalerate, where intraspecific competition favored small individuals by suppressing juvenile growth and increasing adult mortality (Liess and Foit 2010). Our observations indicated that ISI enhanced the effects of Pb exposure in *D. magna*, eliciting significantly lower neonate production across a broad range of Pb exposure concentrations and at two different feeding levels (Fig. 2). Regarding effects thresholds, the Pb exposure had no effect on reproduction at the highest exposure concentration (236 µg/L) in the single-individual exposure whereas the lowest no effect level in the ISI treatment was 25 µg/L representing nearly an order of magnitude increase in sensitivity. Furthermore, the effect of ISI was independent of initial food concentration (Fig. 2), indicating that although total reproduction increased as expected with increasing food availability/quality (Glazier 1992), the increased availability of food resources (between higher and lower algae rations; Fig. 2) did not mitigate the ISI-induced reduction in reproduction within the Pb exposure. Therefore, these results suggest flaws in any hypothesis that experimental scaling has no effect on toxicity outcomes in *D. magna* assays, even when exposure methods are applied to minimized intraspecific competition via normalizing population density and food availability.

### Examining ISI-mediated changes in control reproduction

In non-contaminated exposure conditions, a significant increase in neonate production was observed starting at the lowest level of intraspecific interaction (Fig. 3). *Daphnia* ecologists have also observed ISI-mediated increases in reproductive output as well as changes in other life-history traits in *Daphnia* exposed to water in which conspecifics were reared under crowding stress. Specifically, both Hobæk and Larsson (1990) and Burns (1995) observed increased body size and increased brood size when *D. magn*a were exposed to *D. magna*-conditioned water. Burns (1995) observed increased *D. magna* body length and clutch size in response to exposure to *Daphnia*-conditioned water where the conspecifics experienced high crowding density. In that study, the significantly increased body lengths and clutch sizes in response to the *Daphnia*-conditioned water were initiated at the lowest crowding-treatment level tested (50 animals/L). Our results demonstrated that the presence of just a single conspecific can alter reproductive output in *D. magna* (Fig. 3), although no statistically significant differences for carapace length were observed (results not presented). Although the papers by Hobæk and Larsson (1990) and Burns (1995) demonstrated increased growth and reproduction in *D. magna* experiencing crowding stress-conditioned water, these findings are not universal across studies (see references in the Introduction section of Lürling et al. (2003) where reproduction was reduced in *Daphnia*) and not across *Daphnia* species (Burns 2000). Even within our own study, we observed differences in ISI-mediated effects on neonate production where increased reproduction in controls was observed relative to single animal assays at day 9 whereas single animals within the controls produced more neonates than the ISI-treatment at day 14 (Fig. 2). These results suggest a shift in *D. magna* reproductive strategy; thus, we hypothesize that animals undergoing ISI invest more energy to neonate production earlier on in their life cycle. It is also reasonable to hypothesize that shifting more energy to reproduction as a response to intraspecific competition while exposed to a chemical challenge would increase susceptibility to toxic effects and thus lead to increases in mortality, as observed in Fig. 1 and supported by previous reports that sublethal chemical exposure shifts the energy budget (Coen and Jannssen 2003). Experimental testing of these hypotheses would provide critical advancement in determining the causes of ISI-mediated changes in life history strategy and sensitivity to chemical exposures.

### Daphnia’s “sense” of competition

The results from this study and studies utilizing “*Daphnia*-conditioned water” experiments (cited above) indicate that *D. magna* initiates behaviors and life history strategies characteristic of responses to intraspecific competition when conspecifics are sensed independent of actual population density and food availability. This response is not isolated to *Daphnia*. For example Bédhomme et al. (2005) demonstrated changes in mosquito larvae (*Ades aegypti*) life history traits and population performance comparing larvae grown in clean water to larvae raised in water conditioned by a previous round of conspecific growth. In this example, *A. aegypti* that sensed the presence of conspecifics elicited individual and population-level responses in the absence of actual intraspecific competition. Taken in total, the observations described in this paper indicate that the “sense” of intraspecific competition may be just as important as true competition in affecting life-history strategies.

The ability to sense and react to the presence of conspecifics is the subject of on-going research. For example, Yamada et al. (2010) identified a specific pheromonal signaling mechanism in *Caenorhabditis elegans* that controls density-dependent dispersal behavior which operates by leveraging an endogenous peptide signaling pathway. The effects of substances released during crowding stress in *Daphnia* have been characterized (Lürling et al. 2003), but the effective pheromones/metabolites have not been identified. Alternatively, investigations of *Daphnia*’s inducible defense responses to kairomones (chemical signals of predators), have shown that *Daphnia* respond primarily to the presence of eaten or decomposed conspecifics rather than chemicals specific to the predator (Stabell et al. 2003; Pohnert et al. 2007). Regardless of the mechanism, the sensing of conspecifics and the resultant shift in life history strategy in preparation for intraspecific competition has implications for understanding the ecology of *Daphnia* and for understanding the responses of *Daphnia* to contaminant exposure.

### Implications of ISI for the interpretation of *D. magna* toxicity tests

Given that our results refute the assumption that experiment scaling has no effect on toxicity outcomes in *D. magna*, we assert that it is not appropriate to extrapolate results from scale-up experiments to experiments with individuals, such as the *D. magna* standardized reproduction assay (ASTM 2012; OECD 1984). Specifically, results from assays that have been scaled-up in order to provide sufficient tissue for conducting omics studies (e.g. Stanley et al. 2013; Campos et al. 2013; Garcia-Reyero et al. 2009, 2012; Poynton et al. 2007; Shaw et al. 2007) in which results are applied to explain observations in standard single-animal tests should be interpreted with particular caution. In these studies, ISI-mediated life history responses represent potential confounding factors that may be mistakenly attributed to the experimental treatments.

The broader implications of ISI should also be considered within the general context of routine toxicity assessments with *Daphnia*. For example, standardized acute tests for assessing toxicant induced mortality in *D. magna* generally involve group exposures (commonly, 5–10 daphnids per exposure replicate) whereas in standard chronic toxicity assays where reproductive performance is an endpoint, individual daphnids are usually tracked in order to obtain neonate counts per individual (OECD 1984; ASTM 2012; USEPA 2002). The demonstration of ISI effects in the present study, as well as the observations from the “*Daphnia*-conditioned water” exposure studies (i.e. Hobæk and Larsson 1990; Burns 1995, Lürling et al. 2003; Burns 2000), illustrate the potential for ISI to confound standard toxicity assay results. Given the group exposure methods employed in the acute *D. magna* standard methods, ISI is likely a confounding factor that increases the apparent sensitivity of daphnids to toxicant exposure, as we have demonstrated for Cu and Pb (Fig. 1).

As a final consideration, the effects of ISI should be accounted for when models are used to predict population responses to toxicants from data generated from individual daphnid tests (e.g. Martin et al. 2013a, b; Gabsi et al. 2014a, b; Ananthasubramaniam et al. 2015). As we have demonstrated in this study, disregarding ISI within populations may affect the accuracy of model predictions where toxic outcomes have the potential to be underestimated.

## Conclusions

Given our experimental results, we reject the null hypothesis that ISI among *D. magna* neither impacts its life history strategies, nor affects toxicity assessment measures in Cu and Pb exposure experiments. Furthermore, these results, in addition to those of the published “*Daphnia*-conditioned water” experiments refute the assumption that experiment scaling has no effect on toxicity outcomes in *D. magna*. Thus, extrapolation of results from scale-up experiments to experimental assays with single daphnids, such as the *D. magna* standardized reproduction assay, may confound conclusions. The body of evidence described within the present paper suggests that *Daphnia* need only “sense” the presence of conspecifics to modify life-history strategies characteristic of intraspecific competition and to potentially affect their sensitivity to contaminant exposure. Given that *Daphnia* are likely to be in contact with conspecifics and/or the chemical cues left behind by conspecifics in the environment, it is likely that ISI acts as a modulator of *Daphnia’s* life history strategy and may also affect its sensitivity to contaminant exposure. Observations by Gergs et al. (2013) that contaminant-induced changes in *D. magna* population demography in predator–prey systems can increase the vulnerability of populations to localized extinction demonstrates the need to better understand the implications of ISI in the broader context of *Daphnia* ecology. More research is needed to assess the prevalence of ISI-enhanced toxicity across multiple contaminant classes/modes of toxic action within *D. magna* and determine how widely the phenomenon of ISI-enhanced toxicity extends to species beyond *Daphnia* and the limited collection of other organisms for which it has been observed.

## Electronic supplementary material

Below is the link to the electronic supplementary material.

Supplementary material 1 (DOCX 63 kb)
