# Supplementary material for: Daphnia magna’s sense of competition: intra-specific interactions (ISI) alter life history strategies and increase metals toxicity
Source: Ecotoxicology. 2016 May 5;25:1126–35. doi: 10.1007/s10646-016-1667-1 (PMC4921107; doi:10.1007/s10646-016-1667-1)
Supplement: Supplementary file 1 — Supplementary material 1 (DOCX 63 kb) [file 10646_2016_1667_MOESM1_ESM.docx]

**Supplemental Materials**

**Title: *Daphnia magna’s* Sense of Competition: Intra-Specific Interactions (ISI) Alter Life History Strategies and Increase Metals Toxicity**

Authors: Kurt A. Gust^1*^, Alan J. Kennedy^1^, Nick Melby^1^, Mitchell S. Wilbanks ^1^, Jennifer Laird^1^, Barbara Meeks^2^, Erik B. Muller^3^, Roger M. Nisbet^4^, Edward J. Perkins^1^

Author Affiliations:

^1^US Army, Engineer Research and Development Center, Environmental Laboratory, Vicksburg, MS

^2^SpecPro Technical Services, San Antonio, TX

^3^University of California, Santa Barbara, Marine Science Institute, Santa Barbara, CA

^4^University of California, Santa Barbara, Department of Ecology, Evolution & Marine Biology, Santa Barbara, CA

*Corresponding Author

Phone: 601-634-3593

E-mail: [kurt.a.gust@usace.army.mil](mailto:kurt.a.gust@usace.army.mil)

**SUPPLEMENTAL TEXT**

**MATERIALS AND METHODS**

***Effect of Feeding Methods and Ration on* D. magna *Reproduction***

As part of our overall research effort with *D. magna*, we were interested in the effects of food type and quantity on growth and reproduction. Specifically, in a previous paper, we employed algae only feed at multiple rations to assist in development of a bioenergetics model for predicting *Daphnia* growth and reproduction (Ananthasubramaniam et al. 2015). In these assays *D. magna* were provided an algae-only feeding ration to enable quantifiable feeding measurements for model parameter estimates. In the present effort, we conducted an experimental exposure to test the effects of the algae-only feeding method compared to the standard method of feeding which includes both algae (*Raphidocelis subcapitata*, formerly *Selenastrum capricornutum*) and yeast-cerophyl-trout chow (YCT) as prescribed in the standard ASTM test method (ASTM 2012). The 14 day assay was adapted from Organisation for Economic Cooperation and Development method 202 (OECD 1984). However, since the OECD method does not prescribe defined feeding rations, feeding methods were adapted from ASTM (2012). The lower (1x) feeding ration provided 1.6 X 10^5^ cells/mL and the higher (2x) treatment doubled the ration of algal food to 3.6 X 10^5^ cells/mL. A time series of cumulative reproduction was quantified to inform the assay protocols used in the present study.

**RESULTS**

***Effect of Feeding Methods and Ration on* D. magna *Reproduction***

The algae plus YCT feeding regime had the greatest cumulative neonate production significantly diverging from both the lower (1x) and higher (2x) algae-only treatments at day 7, while the 2x algae-only feeding regime produced the second most neonates diverging from the 1x algae-only ration at day 10 (Supplemental Figure S1). Specifically targeting the 14 day time point, the 1x algae + YCT ration resulted in significantly more neonates than the other rations and the 2x algae-only ration had significantly greater neonates than the 1x algae-only ration. Although, the algae-only feeding method did not provide as high a magnitude of reproduction as the algae + YCT method, it did provide sufficient resolution for identifying differences in reproduction given our treatments of interest in the main study (see main text), as well as providing the advantage of bioenergetics modeling for our future efforts. Regarding the exposure duration, since 14 days provided an adequate exposure period for control animals to produce at least three broods and was sufficient to capture significant differences between our feeding rations of interest, this testing duration as described in OECD 202 (1984) was used for the remainder of the assays described in the main text of this manuscript.

**REFERENCES**

Ananthasubramaniam B, McCauley E, Gust KA, Kennedy AJ, Muller EB, Perkins EJ, Nisbet RM (2015) Relating sub-organismal processes to ecotoxicological and population end points using a bioenergetic model. Ecol Appl 25(6):1691-1710

American Society for Testing and Materials (ASTM) (2012) Standard guide for conducting *Daphnia magna* life-cycle toxicity tests, Method E1193 - 97, West Conshohocken, PA

Organisation for Economic Co-operation and Development (OECD) (1984) *Daphnia* sp. acute immobilization test and reproduction test. Method 202. OECD guideline for testing of chemicals

**SUPPLEMENTAL FIGURES**

**Figure S1.** Cumulative reproduction in Daphnia magna given standard and alternative feeding regimes. Data not sharing the same letter designation were statistically significantly different at a given timepoint. Letters are color coded to correspond with each treatment. Statistical comparisons are provided for day 7 (the first point of significant divergence between standard and alternative feeding rations), day 10 (the first point of significant divergent between the 2x and 1x algae-only feeding rations), day 14 (the testing duration used for the study described in the main manuscript) and day 21 (the standard testing duration).

**Figure S2.** The figure provides a graphical representation of the statistical approach employed in events where the assumptions of the multi-way ANOVAs (2-way in the Cu exposure or 3-way in the Pb exposure) were not met. The example provided represents the three treatments investigated in the Pb experiment, but this design can also be applied to the Cu exposure by removing the “food level” treatment and replacing the Pb exposure concentrations with those conducted in the Cu experiment. Given the completely randomized design and factorial treatment arrangement, one-way ANOVAs with pairwise comparisons were run to test if the ISI and food level treatments affected the endpoints (survival or reproduction) across dose series, as depicted in the horizontal red ovals. Additionally, the effects of the ISI and food level treatments on these endpoints were tested using one-way ANOVA and multiple pairwise comparisons within each individual chemical exposure level as depicted in the vertical red ovals. Overall, the results of the pairwise comparisons allowed us to differentiate the effects of the three treatment levels throughout the entire exposure matrix. We recommend that this supplemental figure is kept in mind when interpreting the experimental results and figures in the main text.
